# *In vitro* digestion and fermentation of jujube-derived polysaccharide PZMP3 and its modulatory effect on gut microbiota and metabolic pathways^☆^

**DOI:** 10.1016/j.fochx.2025.103316

**Published:** 2025-11-29

**Authors:** Xiaoqiong Li, Ke Jiang, Huowang Zheng, Chenyu Zhao, Jinjun Li, Yuling Li, Xiangyu Bian, Jun Du, Liang Chen, Zhigang Zhu, Li Wang, Xiaolong Ji, Hongzhou Cui

**Affiliations:** a State Key Laboratory for Quality and Safety of Agro-products, Institute of Food Science, Zhejiang Academy of Agricultural Sciences, Hangzhou 310021, Zhejiang, PR China; b Department of Dermatology, The First Hospital of Shanxi Medical University, Taiyuan 030000, Shanxi, PR China; c College of Food and Bioengineering, Zhengzhou University of Light Industry, Zhengzhou 450001, Henan, PR China; d Zhejiang Key Laboratory of Intelligent Food Logistic and Processing, Zhejiang Academy of Agricultural Sciences, Hangzhou 310021, Zhejiang, PR China; e Nutrilite Health Institute, Amway (Shanghai) Innovation & Science Co., Ltd., Shanghai 201203, PR China

**Keywords:** Jujube polysaccharides, *In vitro* digestion and fermentation, Gut microbiota modulation, Short-chain fatty acids, Bile acid metabolism

## Abstract

This study evaluated the digestion and fermentation characteristics of PZMP3, polysaccharide rich in galacturonic acid purified from *Z. jujuba cv. Muzao*, and its modulatory effects on the gut microbiota and associated metabolites. *In vitro* simulated gastrointestinal digestion confirmed that PZMP3 resisted enzymatic hydrolysis in the upper gastrointestinal tract and reached the colon intact. During subsequent fecal fermentation with human microbiota (using inulin as a positive control), PZMP3 was degraded by gut microbiota, leading to a gradual reduction in pH and a substantial increase in short-chain fatty acids (SCFAs), reaching a total concentration of 142.06 ± 0.58 mmol/L at 48 h. After digestion and glycolysis, the overall trend of gases shows an increase, while H_2_S is inhibited. Microbiota analysis revealed that PZMP3 selectively promoted the proliferation of *Firmicutes* and *Bifidobacterium* while *Klebsiella* and *Bacteroides* are inhibited. Metabolomic profiling further demonstrated enriched pathways related to bile acids and amino acids, indicating its potential role in regulating host metabolic homeostasis. These findings could highlight the potential of PZMP3 as a gut-targeted functional ingredient for improving intestinal health.

## Highlights

• Experimental design of *in vitro* fermentation of PZMP3.
• Evaluate the dynamics of pH, gases, SCFAs, gut microbes and metabolites.
• Correlate and analyze the effects of gut microbes and metabolites.

## Graphical abstract

**Figure:**
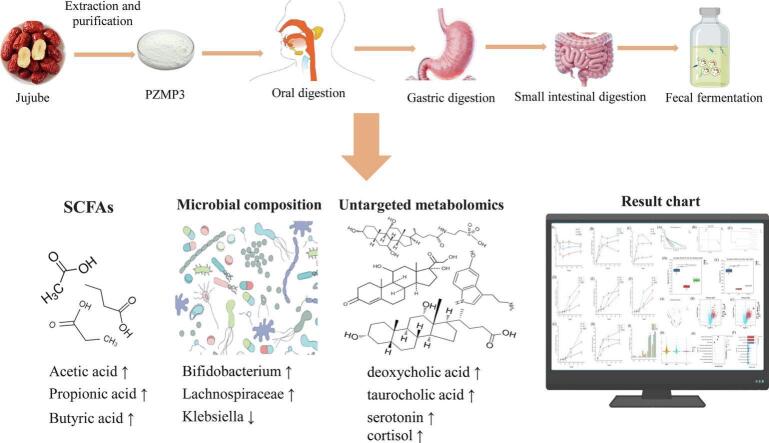

## 1 Introduction

*Ziziphus jujuba* Mill. (jujube), also known as Chinese red date, has been cultivated and consumed in Asia for centuries as both a food and traditional remedy (Kininmonth et al., 2025). Among its diverse bioactive compounds, polysaccharides are considered the major functional components (Ji et al., 2017). These macromolecules usually display complex and heterogeneous structures, composed of monosaccharides such as arabinose, galactose, rhamnose, glucose, and galacturonic acid (Ji, Hou, Yan, et al., 2020). The growing evidence reveals that jujube polysaccharides possess a variety of health-promoting properties-including antioxidant, immunomodulatory, anti-inflammatory, hepatoprotective, anticancer activities, and the modulation of gut microbiota (Ruan et al., 2022). As non-digestible carbohydrates, they could reach the colon intact, where they undergo microbial fermentation, consequently giving rise to short-chain fatty acids (SCFAs) and other metabolites; these compounds are key to governing both the metabolic activity and immune status of the host (Nielsen et al.,2016). Previous studies have demonstrated that prebiotic polysaccharides, including those from jujube fruit, could shift the balance of intestinal microbial communities, decrease the *Bacteroidetes*-to-*Firmicutes* ratio, while also increasing the production of SCFAs like acetate, propionate, and butyrate, thereby supporting intestinal and systemic health (Ji et al., 2024).

Plant-derived polysaccharides have attracted increasing scientific interest owing to their physiological functions and potential as dietary modulators of gut health. Unlike digestible starches, these non-starch polysaccharides generally resist enzymatic hydrolysis in the upper gastrointestinal tract and are delivered to the colon, where they become important substrates for gut microbial fermentation. During this process, specific bacterial groups utilize plant polysaccharides as carbon sources, leading to shifts in microbial composition and the generation of bioactive metabolites, most notably SCFAs. A polysaccharide's fermentability and prebiotic capacity are heavily shaped by its structural features, which include monosaccharide composition, glycosidic linkages, branching degree, and molecular weight (Guo et al., 2025). To illustrate, studies have shown that burdock- and ginseng-derived polysaccharides could selectively boost the growth of *Bifidobacterium* and *Lactobacillus,* while lowering the abundance of pathogenic groups including *Klebsiella* and *Escherichia-Shigella* (Li et al., 2024; Xu et al., 2025). The resulting SCFAs-primarily acetate, propionate, and butyrate-play critical roles in lowering intestinal pH, reinforcing epithelial barrier integrity, modulating host immune responses, and contributing to systemic energy metabolism. Beyond SCFAs, recent metabolomic studies have highlighted that microbial fermentation of plant polysaccharides could also influence bile acid metabolism, a pathway closely linked to lipid absorption, glucose homeostasis, and host immune regulation. Polysaccharides from jujube and areca have been shown to enrich secondary bile acids such as deoxycholic acid and ursodeoxycholic acid, suggesting their potential involvement in host metabolic regulation through the gut-liver axis (Ji et al., 2024; Ji et al., 2025).

*In vitro* models of gastrointestinal digestion and colonic fermentation are widely applied to investigate the functional properties of dietary polysaccharides (Guan et al., 2022). Although several plant polysaccharides have been characterized using these approaches, information on the digestive fate and microbial utilization of jujube polysaccharides with higher galacturonic acid content remains limited. In particular, their resistance to enzymatic hydrolysis, subsequent fermentation dynamics by gut microbiota, and the resulting influence on microbial community structure and metabolic output have not been systematically elucidated. To bridge this gap, the present study focused on PZMP3, a polysaccharide with high galacturonic acid content, isolated from *Ziziphus jujuba cv. Muzao*. Its stability under simulated gastrointestinal conditions was evaluated, followed by *in vitro* fecal fermentation to assess microbial degradation, SCFAs production, and modulation of gut microbiota composition. Furthermore, untargeted metabolomic profiling was performed to elucidate metabolic alterations. These results are expected to provide mechanistic insights into the prebiotic potential of jujube polysaccharides and support their application as gut microbiota-targeted functional ingredients.

## 2 Materials and methods

### 2.1 Chemicals and materials

Fresh fruit of *Z. jujuba cv. Muzao* were collected from the Experimental Orchard on the Loess Plateau. Porcine α-amylase, pepsin, pancreatin, and bile salts were obtained from Sigma-Aldrich (St. Louis, MO, USA). Both short-chain fatty acid (SCFA) standards and solvents for chromatographic and metabolomic analyses were obtained from Aladdin (Shanghai, China). All other chemicals met analytical grade specifications.

### 2.2 Extraction and purification of PZMP3

With minor modifications to a previously described protocol, PZMP3 was extracted and purified (Ji, Yan, Hou, et al., 2020). In short, oven-dried jujube fruits without seeds were ground into powder and subjected to defatting with 80 % ethanol. The residue obtained was extracted using distilled water; the filtrate was concentrated under reduced pressure, and then precipitated by adding four volumes of ethanol, with the process carried out at 4 °C overnight. The precipitate was redissolved in water, and the Sevag method was used for deproteinization. Additional purification steps included an anion-exchange chromatography on a DEAE-Sepharose Fast Flow column (eluted with 0.3 M NaCl) and subsequent gel filtration on a Sephacryl S-300 column. The primary polysaccharide fraction, designated PZMP3, was collected and freeze-dried. PZMP3, a type of polysaccharide, possesses the following characteristics: weak to moderate anionicity (derived from carboxyl groups), a molecular weight range of 50–500 kDa with a narrow distribution, classification as a heteropolysaccharide (containing uronic acids and neutral sugars), moderate branching and water solubility.

### 2.3 Simulated gastrointestinal digestion

*In vitro* digestion was conducted following the standardized INFOGEST 2.0 protocol, with minor modifications made to the procedure. Simulated salivary fluid (SSF), simulated gastric fluid (SGF), and simulated intestinal fluid (SIF) were freshly prepared, adjusted to the required concentrations, and then used in sequential order (Ji et al., 2024).

#### 2.3.1 Oral phase

A modified protocol-derived from the method described by Zhu et al. (2019), was used to carry out the salivary phase digestion of PZMP3. In detail, 500 mg of PZMP3 powder was dissolved in 2 mL of ultrapure water to create a solution with a 1:4 weight-to-volume (*w*/*v*) ratio. Before initiating the reaction, 1.25× concentrated simulated salivary fluid (SSF) and α-amylase (Type XIII-A, activity≥300 U/mg) were equilibrated at 37 °C. The digestion system was assembled by combining 1.6 mL of preheated SSF, 2 mL of PZMP3 solution, 10 μL of 0.75 M CaCl₂·2H₂O, and 300 μL of α-amylase solution, which yielded a final enzyme concentration of 50 U/mL. Ultrapure water was then added to bring the reaction volume to 4 mL, diluting SSF to its 1× working concentration. Following vortexing for homogeneous mixing, the samples were incubated at 37 °C for 2 min with constant shaking at 100 rpm. The enzymatic reaction was terminated by rapid cooling on ice, and the digesta were recovered through centrifugation at 10,000 ×*g* for 10 min at 4 °C.

#### 2.3.2 Gastric phase

We adopted a protocol modified from Zhu et al. (2019) method, with additional adjustments to fit the experimental conditions of the present study. At the conclusion of the salivary phase, 3.2 mL of simulated gastric fluid (SGF, 1.25× concentrated) was gradually incorporated into the 4 mL oral chyme. The pH of the mixture was meticulously regulated to 3.0 using 1 M hydrochloric acid (HCl) to replicate gastric environments. Subsequently, 2 μL of CaCl₂·2H₂O solution and 266.8 μL of porcine pepsin solution (both pre-equilibrated at 37 °C) were added, and the mixture was stirred thoroughly to ensure even dispersion. Deionized water was then added to adjust the SGF to its 1× working concentration. After porcine pepsin was added, the digestion system-with a total volume of 8 mL-was promptly moved to a thermostatic shaker set at 37 °C. The samples were incubated under constant shaking for 2 h, ensuring the gastric phase digestion reached completion.

#### 2.3.3 Intestinal phase

The intestinal phase digestion was performed following a modified procedure based on Zhu et al. (2019), with adaptations to meet the requirements of this study. After the gastric phase, 8 mL of the chyme was transferred into a clean vessel, and 3.2 mL of simulated intestinal fluid (SIF, 1.25 × concentrated) was gradually added. To replicate intestinal conditions, the pH was adjusted to 7.0 using 5 M sodium hydroxide (NaOH). Next, 1.2 mL of bile extract was added and thoroughly mixed to ensure full integration with the gastric digesta. This was followed by the addition of 16 μL of CaCl₂·2H₂O solution and 2 mL of pancreatin, which were evenly dispersed *via* vigorous mixing. The pH was rechecked and fine-tuned to 7.0 if necessary. Deionized water was then added to adjust the simulated intestinal fluid (SIF) to its final 1× concentration. Immediately after adding the enzymes, the total volume (16 mL) was incubated in a 37 °C thermostatic shaker with constant agitation for 2 h to complete intestinal digestion. The reaction was terminated by heating the mixture in a 70 °C water bath for 5 min to inactivate the enzymes. The digesta were then stored for subsequent dialysis and concentration prior to fermentation trials. For analytical purposes, an aliquot was centrifuged at 8500 rpm for 12 min at 4 °C, and the supernatant was collected and stored at −20 °C for further analysis.

### 2.4 Anaerobic fermentation with human fecal microbiota

Six healthy adult volunteers (20–30 years old, with three males and three females) who had not taken antibiotics over the prior three months provided fresh fecal samples. Equal portions of these feces were pooled and homogenized in phosphate-buffered saline (PBS, pH 7.4) at a 1:10 (*w*/*v*) ratio, and the suspension was passed through sterile gauze to obtain a fecal inoculum (Jiang et al., 2024). During fermentation, 1 mL of the fecal suspension was mixed with 5 mL of sterile fermentation medium. The medium was modified differently for each group: the experimental group was supplemented with digested PZMP3 (final concentration 20 mg/mL), the positive control group was supplemented with inulin (final concentration 20 mg/mL, a well-recognized prebiotic), and the blank control group was without any added carbohydrate. The mixtures were subjected to anaerobic incubation (95 % N₂, 5 % H₂) at 37 °C, and samples were harvested at 0, 6, 12, 24, and 48 h. Following centrifugation at 8000 rpm for 10 min, the supernatants were utilized for pH measurement and SCFAs determination, whereas the pellets were preserved at −80 °C to be used later for microbiota and metabolomic analyses.

### 2.5 Quantification of total and reducing sugars

Total carbohydrate levels were quantified using the phenol‑sulfuric acid assay, employing glucose for calibration purposes, while reducing sugars were measured with the dinitrosalicylic acid (DNS) assay (Ji et al., 2020). Absorbance was recorded at 490 nm and 540 nm on a UV–Vis spectrophotometer respectively, and results were expressed as glucose equivalents. Standard curves were prepared from freshly made glucose solutions, and all assays were carried out in triplicate. These analyses were used to monitor changes in sugar content during digestion and fermentation.

### 2.6 pH and gas measurement

The pH of the fermentation broth was monitored at designated time points using a calibrated pH meter. Gas composition, including CO₂, H₂, CH₄, and H₂S, was determined with a portable gas analyzer (manufacturer's protocol). At each sampling interval (0, 6, 12, 24, and 48 h), 0.5 mL aliquots of fermentation broth were withdrawn with sterile syringes. Each sample's pH was determined immediately, with the measured values documented to be used in subsequent analysis. Prior to gas analysis, the system was flushed to remove residual gases. Fermentation vials were then placed in the analyzer chamber, and headspace concentrations of CO₂, H₂, CH₄, and H₂S were quantified (Ji et al., 2024).

### 2.7 Determination of SCFAs

Calibration standards for SCFAs analysis were prepared in two steps (Tian et al., 2024). Initially, stock solutions of acetic acid and propionic acid (200 mL each) were accurately measured and placed in sterile containers. These were then combined with a pre-mixed solution containing isobutyric acid, n-butyric acid, isovaleric acid, and n-pentanoic acid in 10-mL volumetric flasks, with acetic and propionic acids added in sequence. To achieve homogeneity, 1 mL of ultrapure water was added to each flask containing the six SCFAs. Fermentation broths were centrifuged at 8000 rpm for 10 min, and the resulting supernatants were transferred to sterile tubes and stored at −20 °C until analysis. Prior to chromatographic determination, samples were clarified *via* an additional centrifugation step at 12,000 rpm for 10 min. SCFAs quantification was conducted using a Shimadzu QP2010 gas chromatograph fitted with a DB-FFAP capillary column (60 m × 0.25 mm × 0.5 μm, Agilent Technologies, Santa Clara, CA, USA). Helium was used as the carrier gas at a constant flow rate of 0.7 mL/min. The oven temperature protocol was as follows: initial hold at 50 °C for 1 min, a ramp of 15 °C/min to 240 °C, and an isothermal phase at 240 °C for 10 min to ensure all analytes eluted completely. SCFA concentrations were derived from calibration curves prepared with the corresponding standards.

### 2.8 Microbial community analysis

Sediment samples collected after 48 h of fermentation were submitted to Majorbio Bio-Pharm Technology Co., Ltd. (Shanghai, China) for 16S rRNA gene sequencing. Amplification of the bacterial V3-V4 hypervariable regions was performed on an ABI GeneAmp® 9700 thermal cycler (Applied Biosystems, Foster City, CA, USA). PCR products were purified using a DNA gel extraction kit (Axygen Biosciences, Union City, CA, USA), quantified *via* a QuantiFluor-ST fluorometer, and sequenced on the Illumina MiSeq platform according to the manufacturer's instructions. Raw reads were deposited in the NCBI Sequence Read Archive (accession no. PRINA935523). The sequencing data were demultiplexed, quality-filtered, and merged to generate high-quality tags. Amplicon sequence variants (ASVs) were inferred using the DADA2 pipeline, and representative sequences were taxonomically assigned against the SILVA 138/16S rRNA database with a confidence threshold of 0.7. This process yielded high-resolution microbial profiles for subsequent community analysis (Ji, Hou, Gao, et al., 2020).

### 2.9 Untargeted metabolomics analysis

Following 48 h of *in vitro* fermentation, metabolite extraction was performed by mixing 400 μL of supernatant with 1.2 mL of pre-chilled methanol-acetonitrile (1,1, *v*/v). The mixture was vortexed for 30 s, sonicated at 40 kHz for 30 min, and held at −20 °C for 3 h to facilitate protein precipitation. Subsequent centrifugation at 10,000 ×*g* for 10 min at 4 °C was carried out, and the supernatant was collected. Of this supernatant, 750 μL was transferred to LC vials for UPLC-MS/MS analysis, and 50 μL was kept aside for system quality control. Chromatographic separation was achieved using a Waters ACQUITY UPLC BEH C18 column (2.1 × 100 mm, 1.7 μm) at 40 °C. A binary mobile phase-consisting of (A) 0.1 % formic acid in 2 % acetonitrile and (B) 0.1 % formic acid in 98 % acetonitrile was delivered at a flow rate of 0.4 mL/min, with an injection volume of 5 μL. Mass spectrometry parameters were adjusted to ensure broad coverage of metabolites (Yu et al., 2023).

### 2.10 Statistical analysis

All data are presented as mean ± standard deviation (SD). Group differences were assessed *via* one-way analysis of variance (ANOVA), and pairwise comparisons were conducted using Duncan's multiple range test. Statistical computations were carried out with SPSS 27.0 software (IBM, Armonk, NY, USA), where a *P*-value <0.05 was considered indicative of a significant difference. Figures and graphs were created using Origin 2021 (OriginLab, Northampton, MA, USA). Correlation analysis between gut microbes and metabolites was performed using Spearman's rank correlation coefficient, and multiple testing correction was applied using the false discovery rate (FDR) method, with an adjusted *P*-value <0.05 considered statistically significant.

## 3 Results and discussion

### 3.1 Reducing and total sugar changes during *in vitro* digestion and fermentation

To evaluate the susceptibility of PZMP3 to enzymatic hydrolysis and microbial utilization, total and reducing sugar levels were monitored during simulated gastrointestinal digestion and subsequent colonic fermentation (Table S1). Following digestion, the total sugar concentration of PZMP3 was 0.220 ± 0.007 mg/mL, whereas reducing sugars reached 1.306 ± 0.020 mg/mL. After 24 h of fecal fermentation, both indices declined markedly to 0.006 ± 0.001 mg/mL and 0.863 ± 0.008 mg/mL, respectively (*P* < 0.05). The sharp decrease in total sugar content, together with the more moderate reduction in reducing sugars, indicates that polysaccharide backbones were extensively cleaved, releasing smaller carbohydrate fragments that were gradually consumed by intestinal microbiota (Ye et al., 2023).

Interestingly, the persistence of reducing sugars during fermentation suggests that hydrolysis intermediates (*e.g.*, oligosaccharides and monosaccharides) were generated faster than they were metabolized, thereby providing a continuous substrate supply for microbial growth (He et al., 2025). Similar patterns have been reported for other non-starch polysaccharides, such as inulin and burdock polysaccharides, which also release fermentable saccharides that sustain microbial activity over time (Li et al., 2024). These findings support the notion that PZMP3 is a highly fermentable substrate and might act as an effective energy source for gut bacteria, thereby contributing to the modulation of microbial community structure and metabolic output.

### 3.2 PZMP3 *in vitro* fermentation on the influence of pH and SCFAs

**Fig. 1:**
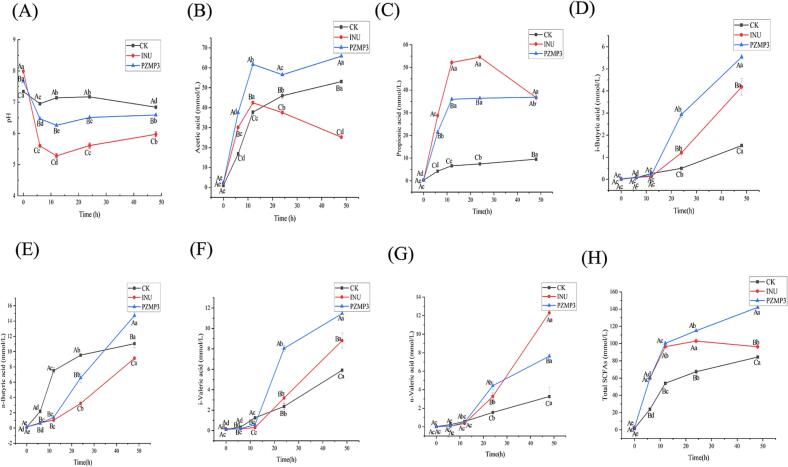
Changes in pH, SCFAs, and gas production during fermentation. Variations of (A) pH, (B) acetic, (C) propionic, (D) i-butyric, (E) n-butyric, (F) i-valeric, (G) n-valeric and (H) total SCFAs. Superscript A-D indicates significant differences between different groups at the same time point (*P* < 0.05), superscript a-d indicates significant differences at different times within the same group (*P* < 0.05).

The pH is an important indicator of microbial activity during fermentation and reflects the extent of carbohydrate decomposition by gut microbiota (Zhang, Xiao, et al., 2023). A gradual acidification of the fermentation system is generally considered beneficial for host intestinal health, as lower pH values favor the proliferation of commensal bacteria while restricting opportunistic pathogens (Sun et al., 2024). As shown in Fig. 1A, the inulin (INU) group exhibited the most pronounced pH decline (*P* < 0.05), decreasing from 7.99 to 5.60 within the first 6 h. PZMP3 also caused a significant reduction in pH, from 7.67 to 6.74, although the magnitude of change was smaller. This moderate acidification might be related to the more complex and branched structure of PZMP3 compared with the water-soluble inulin, consistent with the observations of Zhang et al. (2024). The observed pH decrease likely reflects the conversion of polysaccharides into organic acids, which in turn helps maintain an intestinal environment favorable for beneficial microbiota.

The SCFAs are the major end-products of microbial fermentation of indigestible carbohydrates and serve as crucial mediators linking dietary polysaccharides to host health (Palmnas-Bedard et al., 2022). As illustrated in Fig. 1B-H, SCFA concentrations increased steadily during the fermentation process in all groups. Notably, PZMP3 supported the most extensive SCFAs production, with total levels rising from 2.94 ± 0.04 mmol/L at baseline to 142.06 ± 0.58 mmol/L at 48 h, significantly higher than those of both the CK and INU groups (*P* < 0.05). In contrast, the CK and INU groups reached 84.38 ± 0.37 mmol/L and 96.33 ± 2.51 mmol/L, respectively. These results demonstrate that PZMP3 is a highly fermentable substrate with strong potential to stimulate microbial metabolism.

Further compositional analysis revealed that acetate, propionate, and butyrate were the predominant SCFAs generated from PZMP3 fermentation, reaching 65.85 ± 0.14, 36.88 ± 0.04, and 14.72 ± 0.40 mmol/L at 48 h respectively. Each of these metabolites contributes differently to host physiology: acetate regulates lipid and glucose metabolism, propionate promotes epithelial proliferation and improves insulin sensitivity, while butyrate functions as the primary energy source for colonocytes and exhibits potent anti-inflammatory properties (Geng et al., 2023; Zhang et al., 2020). Interestingly, in the INU group, acetate and propionate concentrations declined after 24 h, suggesting that specific microbial populations may have further metabolized these intermediates, possibly *via* lactate-to-butyrate conversion pathways as reported previously (Zhang, Hu, et al., 2022). In contrast, PZMP3 sustained the accumulation of all three major SCFAs throughout the fermentation cycle, indicating a more stable release of fermentable substrates. The SCFAs of the INU group peaked at 12 h and then stabilized, while PZMP3 continued to slowly increase after 12 h, indicating that inulin is rapidly digestible prebiotics, while PZMP3 a slowly fermented prebiotic.

The slowly fermented property of PZMP3 may be attributed to its specific structural features. PZMP3 was a heteropolysaccharide rich in galacturonic acid (accounting for 45 % of total monosaccharides) with moderate branching and a molecular weight range of 50–500 kDa. Galacturonic acid residues in PZMP3 were linked primarily by α-(1 → 4) glycosidic bonds, which require specific microbial enzymes (*e.g.*, polygalacturonases, pectin lyases) for cleavage. These enzymes were produced by only a subset of gut microbes (*e.g.*, certain strains of *Bifidobacterium*, *Lachnospiraceae*), and their relatively low abundance in the fecal inoculum may limit the rate of PZMP3 degradation. In contrast, inulin, a linear fructan linked by *β*-(2 → 1) glycosidic bonds, could be hydrolyzed by a broader range of gut microbes expressing fructanases, leading to more rapid fermentation. Additionally, the moderate branching of PZMP3 may further hinder the access of microbial enzymes to glycosidic bonds, contributing to its slow fermentation kinetics.

Taken together, these findings delineate distinct fermentation kinetics: inulin, as a rapidly fermented prebiotic, spurred a sharp rise in SCFAs that peaked at 12 h and then plateaued. In contrast, PZMP3 was degraded gradually and fermented slowly yet persistently by the gut microbiota, leading to a continuous increase in SCFAs beyond 12 h and thus maintaining higher levels in the later stages. This slow-digesting property highlights the potential of PZMP3 as a sustained-release prebiotic for promoting intestinal health and metabolic regulation.

### 3.3 Gas production profiles during *in vitro* fermentation

**Fig. 2:**
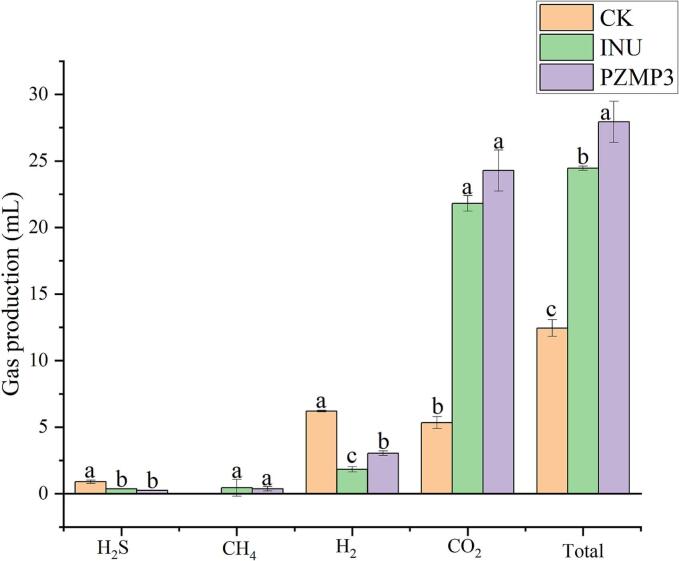
Yield of each gas at 6 h of fermentation. Different letters in the table indicate significant differences (*P* < 0.05).

Gas formation serves as an important marker of microbial activity and substrate fermentability during colonic fermentation (Wu et al., 2022). As shown in Fig. 2, the three groups displayed distinct gas profiles after 6 h of incubation. In the absence of fermentable carbohydrates, the CK group produced the lowest overall gas volume (12.45 ± 0.62 mL), primarily consisting of H₂ (6.21 ± 0.06 mL) and CO₂ (5.34 ± 0.45 mL). Notably, this group exhibited relatively high H₂S production (0.90 ± 0.11 mL), indicating increased microbial metabolism of proteins or sulfur-containing substrates when carbohydrates were scarce (Blachier et al., 2021). In contrast, both inulin and PZMP3 markedly enhanced gas generation (*P* < 0.05). Inulin fermentation yielded 24.46 ± 0.16 mL of total gas, dominated by CO₂ (21.81 ± 0.58 mL), reflecting its rapid solubilization and preferential utilization by saccharolytic bacteria. However, H₂ production was lower in the INU group (1.83 ± 0.19 mL) compared with PZMP3 (3.04 ± 0.17 mL), suggesting differences in fermentation pathways activated by the two substrates. Among all treatments, PZMP3 supported the highest gas output (27.94 ± 1.55 mL), with elevated CO₂ (24.28 ± 1.55 mL) and H₂ (3.04 ± 0.17 mL), indicating vigorous microbial metabolism and efficient carbohydrate degradation. Small amounts of CH₄ were detected in the INU (0.44 ± 0.63 mL) and PZMP3 (0.37 ± 0.17 mL) groups but not in CK, while the lowest H₂S accumulation was observed with PZMP3 (0.24 ± 0.01 mL), pointing to a shift toward saccharolytic rather than proteolytic fermentation.

These results suggest that PZMP3 promoted robust microbial activity comparable to or exceeding that of inulin, while simultaneously suppressing H₂S formation. Reduced H₂S is particularly relevant, as excessive production of this metabolite has been associated with mucosal damage and intestinal disorders (Mi et al., 2025). By favoring carbohydrate-driven fermentation over amino acid catabolism, PZMP3 might contribute to a healthier colonic environment. This dual effect-stimulating gas metabolism while minimizing harmful by products-highlights PZMP3 as a promising prebiotic candidate with distinct advantages over conventional substrates.

### 3.4 PZMP3 effects on microbial communities

#### 3.4.1 PZMP3 effects on microbial diversity

**Fig. 3:**
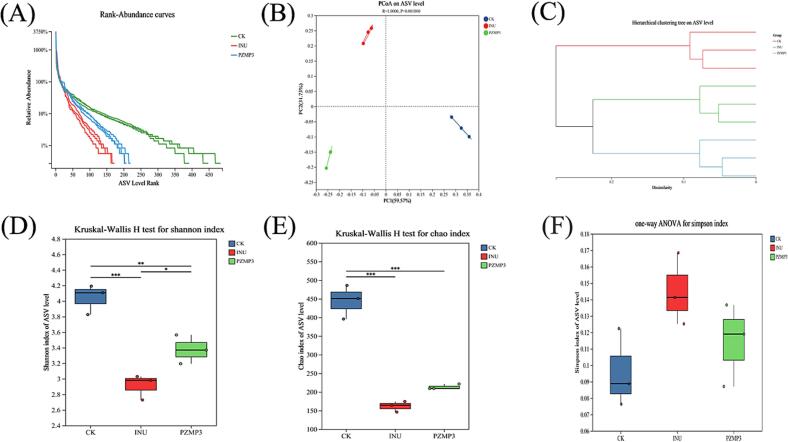
(A) Rank abundance curves, (B) PCoA analysis, (C) hierarchical clustering tree, (D) shannon indexes, (E) Chao indexes and (F) Sinmpson indexes. “*” and “***” indicate levels of statistical significance for the comparisons among groups. “*” means *P* < 0.05. “***” means *P* < 0.001.

The influence of PZMP3 on gut microbial structure during *in vitro* fermentation was assessed using 16S rRNA gene sequencing. Species accumulation curves indicated adequate sequencing depth across all groups, as the curves gradually plateaued with increasing sample size, suggesting that the microbial diversity was well captured (Tian et al., 2025). Rank-abundance distributions (Fig. 3A) showed clear differences in richness and evenness: the CK group displayed the highest species uniformity, followed by PZMP3, whereas inulin exhibited the lowest values. This pattern suggests that PZMP3 could alter microbial structure by selectively stimulating certain taxa while reducing overall community diversity.

Community-level differences were further evaluated through *β*-diversity analysis. Principal coordinate analysis (PCoA) based on ASV profiles (Fig. 3B) explained 59.57 % and 31.73 % of the variance along PCoA1 and PCoA2, respectively, and revealed distinct clustering among CK, INU, and PZMP3. PERMANOVA confirmed that these separations were statistically significant. Consistent with this, Bray-Curtis clustering (Fig. 3C) showed that PZMP3 treatment led to a microbial composition that diverged substantially from CK, indicating functional and compositional restructuring of the community.

To further characterize community diversity, α-diversity indices were calculated, including the Chao index (richness), Shannon index (diversity), and Simpson index (dominance) (Li et al., 2021). As shown in Fig. 3D, PZMP3 significantly reduced Shannon and Chao indices compared with CK (*P* < 0.05), suggesting enrichment of specific bacterial populations at the expense of overall diversity. Similar outcomes have been reported for other plant polysaccharides such as *Siraitia grosvenorii* polysaccharides and jujube-derived fractions (Ji et al., 2024; Teng et al., 2025) which also promoted selective microbial proliferation while reducing overall evenness. Interestingly, the Chao index of the PZMP3 group did not differ significantly from inulin (Fig. 3E), implying that although both substrates supported comparable richness, PZMP3 may have modulated the metabolic output by reshaping the abundance of functionally important bacteria within dominant taxa. As Fig. 3F shows Simpson index (biodiversity measure) across CK, INU, PZMP3 groups. INU has the highest index (lowest diversity), CK the lowest, and PZMP3 intermediate. There is no significant difference among the three groups. Reduction in alpha-diversity (Shannon and Chao indices) following PZMP3 treatment, interpreted as a targeted effect, is not uncommon in studies of potent prebiotics. For example, similar trends have been reported for polysaccharides from *Siraitia grosvenorii* and *burdock*, where selective enrichment of beneficial taxa (*e.g.*, *Bifidobacterium*, *Lactobacillus*) was accompanied by a decrease in overall community diversity, without adverse effects on intestinal homeostasis. This suggests that the reduced diversity induced by PZMP3 may reflect a shift toward a more functionally specialized microbial community, rather than a loss of beneficial ecological function.

Taken together, these findings indicate that PZMP3 exerts a targeted effect on gut microbial composition-reducing overall diversity but promoting specific functional groups. Such selectivity may underlie its capacity to drive distinct metabolic profiles, including enhanced SCFAs production, and suggests that PZMP3 could act as a specialized prebiotic with functional outcomes different from those of inulin.

#### 3.4.2 PZMP3 effects on microbial components

**Fig. 4:**
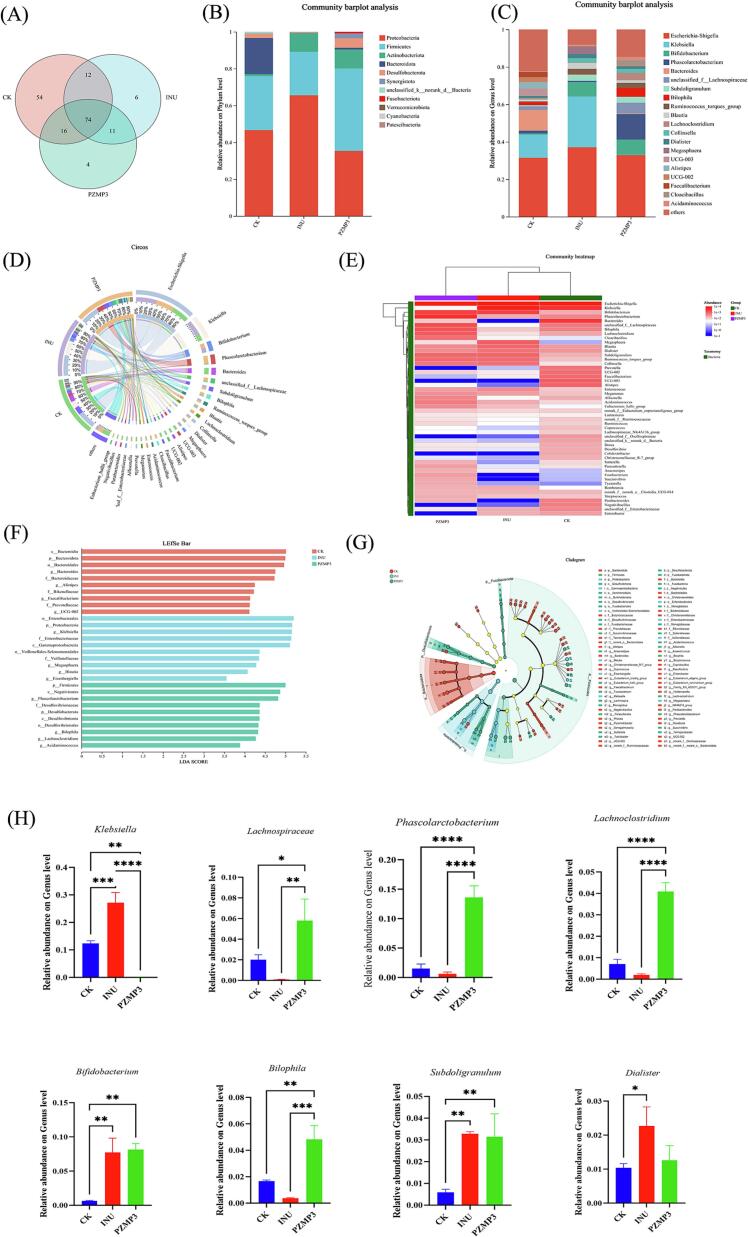
(A) Venn diagram of gut microbiota, The composition of gut microbiota at (B) phylum and (C) genus level, (D) circos analysis, (E) fermentation, (F) LDA score, and (G) Lefse analysis of gut microbiota. The LDA score threshold used for LEfSe analysis is LDA > 2.0.(H) Analysis of differences in microbial communities between groups at the genus level, * indicates *P* < 0.05, ** indicates *P* < 0.005, *** indicates *P* < 0.0001.

The effects of PZMP3 on gut microbial community structure were further explored at multiple taxonomic levels. Venn diagram analysis (Fig. 4A) revealed that 74 amplicon sequence variants (ASVs) were shared among all groups, representing 41.81 % of the total, while the remaining ASVs exhibited group-specific differences. The INU and PZMP3 groups contained 6 and 4 unique ASVs, respectively, suggesting that PZMP3 treatment introduced subtle yet distinct changes in microbial diversity compared with the other groups. At the phylum level (Fig. 4B), *Firmicutes*, *Bacteroidota*, and *Actinobacteria* dominated across all samples. Notably, the PZMP3 group exhibited an increased relative abundance of *Firmicutes* and *Actinobacteria* but a decrease in Bacteroidota compared with CK and INU. An elevated *Firmicutes*-to-*Bacteroidota* (F/B) ratio is often linked to carbohydrate utilization and butyrate production (Liu et al., 2025), and this shift might be associated with the lower pH observed in the PZMP3 fermentation system. The increase in *Actinobacteria*, largely attributed to *Bifidobacterium spp.*, further highlights the prebiotic potential of PZMP3, as these taxa are recognized as key beneficial members of the human gut. At the genus level (Fig. 4C), PZMP3 markedly enriched *Bifidobacterium*, *Lachnospiraceae*, and *Phascolarctobacterium*, while reducing *Klebsiella* and *Bacteroides significantly.* The promotion of *Lachnospiraceae* is particularly relevant, as members of this family could degrade complex polysaccharides to produce SCFAs such as butyrate, which contribute to epithelial barrier integrity and immune modulation (Wang et al., 2025). Several strains have also been reported to suppress colorectal tumor progression, indicating additional health-promoting functions. Similarly, *Phascolarctobacterium* utilizes succinate to generate propionate (Ding et al., 2019), linking PZMP3 intake to enhanced propionate availability. Conversely, the reduction of *Klebsiella* is beneficial, as this opportunistic pathogen is associated with systemic infections and poor clinical outcomes (Dong et al., 2024). Together, these results suggest that PZMP3 selectively fosters beneficial taxa while suppressing potential pathogens, thereby contributing to intestinal homeostasis.

Comparative analysis (Fig. 4D-F) also indicated that PZMP3 induced microbial shifts distinct from inulin. Whereas inulin markedly reduced *Bacteroides* abundance and promoted *Megasphaera*, PZMP3 showed stronger effects on *Firmicutes* and *Bifidobacterium*, suggesting that the two substrates shape microbial metabolism *via* different ecological pathways. Given that *Megasphaera* is linked to lactic acid and butyrate production (Dong et al., 2024), the differential responses highlight that PZMP3 might provide complementary or even superior prebiotic benefits compared with conventional substrates. To identify discriminant taxa, LEfSe analysis was performed (Fig. 4G-H). The class *Clostridia* was significantly enriched under PZMP3 treatment. Members of *Clostridia,* particularly *Clostridium butyricum*, are known for their butyrate-producing capacity and beneficial roles in regulating oxidative stress, enhancing immune function, and supporting gut barrier integrity (Cai et al., 2025; Su et al., 2023). Similarly, *Lachnoclostridium* was identified as a key biomarker in the PZMP3 group. This genus has been linked to metabolic regulation and disease prevention, including obesity and intestinal inflammation, through SCFAs production and modulation of host immunity (Cheng et al., 2022). Importantly, cross-feeding interactions within *Firmicutes* and *Clostridia*-whereby acetate and lactate serve as substrates for butyrate synthesis-might further enhance SCFAs yield and stabilize microbial networks (Zhang, Zhang, et al., 2023). Such interactions, coupled with the acidification induced by fermentation, likely reinforce the dominance of *Firmicutes* while suppressing acid-sensitive competitors.

Collectively, these results demonstrate that PZMP3 not only shifts microbial composition toward beneficial taxa but also strengthens functional metabolic pathways related to SCFAs production and immune regulation. Compared with inulin, PZMP3 appears to exert a broader spectrum of prebiotic effects, underscoring its potential as a functional dietary ingredient for maintaining gut health and preventing metabolic disorders.

### 3.5 PZMP3 effects on gut bacteria fermentation metabolites

**Fig. 5:**
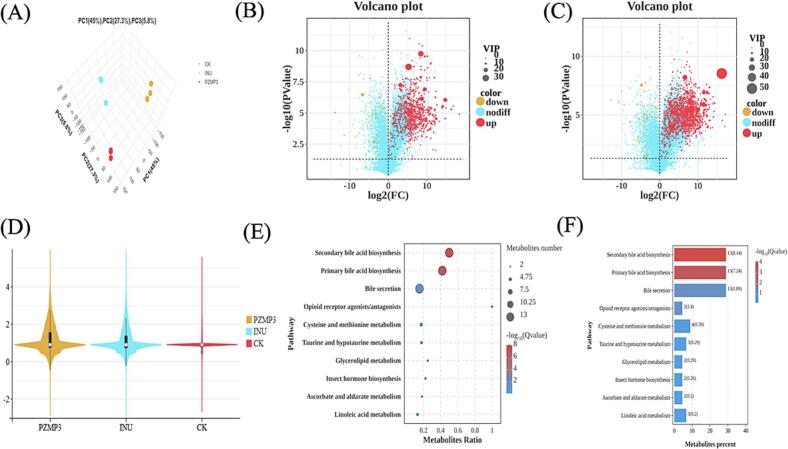
(A) Microbial diversity, diagram of metabolite volcanoes between the two groups in (B) anionic and (C) cationic mode, (D) *β*-diversity, (E) KEGG metabolic pathway diagram, and (F) enrichment bar diagram.

Metabolomic profiling was employed to further elucidate the functional consequences of PZMP3 fermentation and its potential impact on host-microbiota interactions. Using ultra-performance liquid chromatography-tandem mass spectrometry (UPLC-MS/MS), a comprehensive metabolite landscape of the fermentation supernatant was generated. Principal component analysis (PCA) (Fig. 5A) revealed a clear segregation of the PZMP3 and CK groups, with the first three components (PC1, PC2, and PC3) explaining 45.0 %, 27.3 %, and 5.8 % of the variance, respectively. This distinct clustering strongly supports the notion that PZMP3 markedly reshaped microbial metabolic activity compared with the control. Volcano plot analysis further demonstrated profound metabolic reprogramming. Under positive ion mode, 954 metabolites exhibited significant changes, whereas 1587 metabolites were differentially regulated under negative ion mode (Figs. 5B-C). Among these, 1405 metabolites were up-regulated and 182 were down-regulated in the PZMP3 group, underscoring broad activation of microbial metabolic pathways. Importantly, both the PZMP3 and INU groups displayed pronounced elevations in markers associated with microbial abundance and activity (Fig. 5D), highlighting their prebiotic capacity. However, the distinct clustering patterns suggest that PZMP3 and inulin modulate intestinal metabolism through partially divergent mechanisms.

Pathway enrichment analysis revealed that the PZMP3 treatment predominantly affected bile acid metabolism and secretion, pantothenate/CoA biosynthesis, porphyrin metabolism, and secondary bile acid biosynthesis (Figs. 5E-F, Table S2). In particular, bile secretion emerged as one of the most significantly enriched pathways. Several metabolites, including deoxycholic acid, taurocholic acid, serotonin, and cortisol, were upregulated. The observed upregulation of bile acids suggests a potential for PZMP3 to influence host bile acid metabolism, which is closely associated with enterohepatic circulation and bile signaling. Bile acids are increasingly recognized as immunomodulatory molecules capable of regulating inflammasome activation and epithelial renewal, thereby reinforcing intestinal barrier integrity (Zhang, Liu, et al., 2022). Beyond bile metabolism, PZMP3 intervention also altered tryptophan-derived metabolites, which might act as ligands for the aryl hydrocarbon receptor (AHR). Previous studies have linked AHR activation to alleviation of DSS-induced colitis, highlighting its role in maintaining mucosal immune tolerance and attenuating intestinal inflammation (Zhao et al., 2020). These results suggest that PZMP3 could ameliorate inflammation through synergistic mechanisms-by stimulating bile acid secretion and enhancing tryptophan metabolism. Notably, the metabolite profile is consistent with potential FXR activation, which requires further investigation *in vitro* (Zhai, et al., 2025).

The enrichment of pathways such as pantothenate/CoA biosynthesis and porphyrin metabolism also provides insights into PZMP3's potential effects on gut health. Pantothenate is a precursor of CoA, a key cofactor involved in microbial energy metabolism (*e.g.*, fatty acid synthesis and oxidation) and the production of SCFAs. Enhanced pantothenate/CoA biosynthesis may reflect increased microbial metabolic activity, supporting the robust SCFAs production observed in the PZMP3 group. Porphyrin metabolism, on the other hand, is associated with heme biosynthesis, which is essential for the growth and function of certain gut microbes (*e.g.*, obligate anaerobes that require heme as a cofactor for respiratory enzymes). Alterations in porphyrin metabolism may indicate shifts in the composition of microbial taxa dependent on heme, further contributing to the restructuring of the gut microbial community induced by PZMP3.

Taken together, metabolomic evidence indicates that PZMP3 exerts dual benefits: it enhances saccharolytic microbial fermentation and modulates host-relevant metabolic pathways, particularly those involved in bile acid turnover and immune regulation. Compared with the established prebiotic inulin, PZMP3 appears to elicit broader metabolic responses, suggesting potential applications not only in shaping gut microbiota but also in mitigating inflammatory conditions such as colitis. These findings could provide novel insights into the multifunctional role of PZMP3 as a dietary ingredient with both nutritional and therapeutic relevance.

### 3.6 Correlation analysis of gut microbes and metabolites

**Fig. 6:**
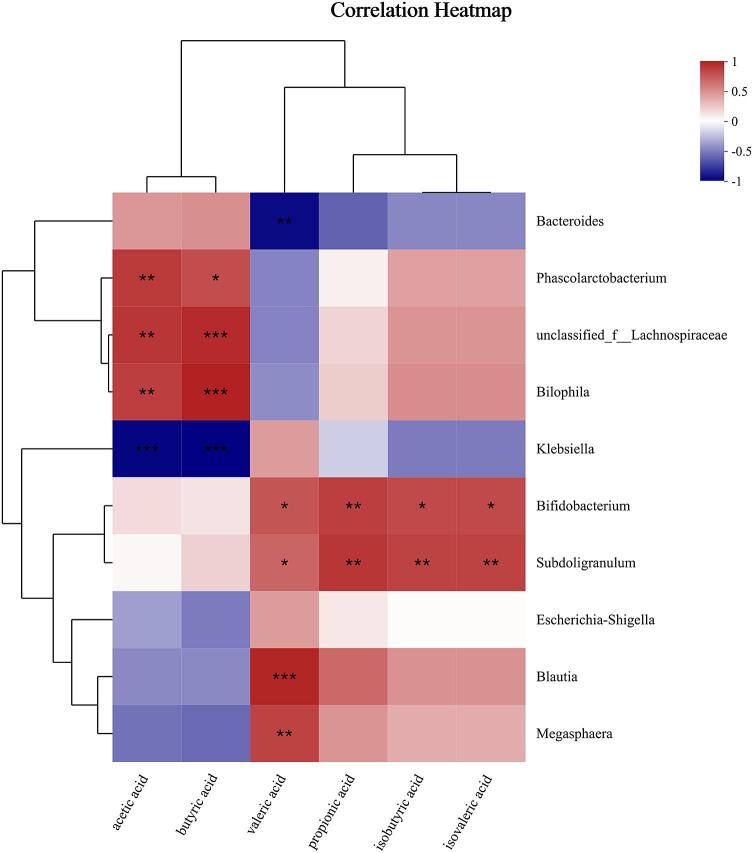
Correlation heatmap of bacterial genera and short-chain fatty acids. Color (red for positive, blue for negative; intensity shows correlation strength) and symbols (* means *P* < 0.05, ** means *P* < 0.01, *** means *P* < 0.001) are used. (For interpretation of the references to color in this figure legend, the reader is referred to the web version of this article.)

Combined 16S rRNA sequencing and metabolomic analyses revealed that PZMP3 profoundly reshaped gut microbial communities and metabolic functions. At the taxonomic level, PZMP3 supplementation increased the relative abundance of *Firmicutes*, especially *Lachnospiraceae* and *Ruminococcaceae*, both recognized as key degraders of complex polysaccharides and major contributors to butyrate production. The elevated *Firmicutes*-to-*Bacteroidota* ratio observed in the PZMP3 group is consistent with the lower pH environment established during fermentation, which generally suppresses *Bacteroidota*. Moreover, PZMP3 reduced potentially pathogenic taxa such as *Klebsiella* while enriching beneficial genera including *Bifidobacterium* and *Phascolarctobacterium*, indicating a selective stimulation of saccharolytic and probiotic populations that contribute to intestinal homeostasis (Song et al., 2021; Xue et al., 2024). The Fig. 6 shows that *Bifidobacterium* and *Subdoligranulum* positively correlate with multiple SCFAs, and the *Klebsiella* negatively correlates with acetic and butyric acids.

These compositional changes were paralleled by distinct metabolic alterations. PZMP3 fermentation produced significantly greater amounts of acetate, propionate, and butyrate than either inulin or the control, highlighting its strong fermentability. In addition to SCFAs, metabolomic profiling showed enrichment of bile acid biosynthesis and secretion, lipid metabolism, and amino acid pathways (*e.g.*, cysteine, methionine, taurine, and hypotaurine). Several bile acids, including cholic acid and ursodeoxycholic acid, were markedly upregulated, while stercobilinogen and cholesterol sulfate were decreased, suggesting that PZMP3 may promote bile acid turnover and cholesterol regulation (Zhang et al., 2025). These metabolic shifts extend the functional scope of PZMP3 beyond carbohydrate fermentation, implicating it in lipid digestion and systemic metabolic balance.

The integrative analysis further reveals that the enriched microbial taxa, particularly *Lachnospiraceae*, may directly drive the observed metabolic shifts. *Lachnospiraceae*, a key member of *Firmicutes*, possesses enzymes such as bile salt hydrolases (BSHs) and 7α-hydroxysteroid dehydrogenases (7α-HSDHs), which are involved in the deconjugation and oxidation of primary bile acids (*e.g.*, cholic acid) to secondary bile acids (*e.g.*, deoxycholic acid). The significant upregulation of secondary bile acids in the PZMP3 group is consistent with the increased abundance of *Lachnospiraceae*, suggesting that PZMP3 may promote bile acid transformation by enriching this bacterial family. Additionally, *Lachnospiraceae* is a major producer of butyrate, and the elevated butyrate levels in the PZMP3 group are tightly linked to the enrichment of this taxa, further confirming the direct role of specific microbes in shaping metabolic profiles. This microbe-metabolite interaction underscores the mechanism by which PZMP3 modulates gut health through selectively enriching functionally important microbes that drive beneficial metabolic changes.

Integrative analysis links these observations: enrichment of *Lachnospiraceae* and *Ruminococcaceae* likely drove SCFAs production, while their involvement in bile acid transformation explains the elevated bile acid metabolites. In contrast, inulin favored classical probiotics such as *Bifidobacterium* and *Lactobacillus*, with metabolic profiles dominated by carbohydrate-associated pathways (Zhang et al., 2025). Collectively, these results suggest that PZMP3 exerts broader metabolic effects than inulin, simultaneously enhancing carbohydrate-derived energy release and regulating lipid-related metabolism. Such dual modulation of microbial composition and metabolite production underscores the potential of PZMP3 as a promising prebiotic candidate with a promising prebiotic candidate with multifunctional potential, with implications for gut health, metabolic regulation, and immune function. However, this needs to be validated in future *in vivo* studies.

## 4 Conclusion

In summary, this study demonstrates that jujube-derived polysaccharide PZMP3 is efficiently fermented by human gut microbiota, leading to selective enrichment of beneficial taxa such as *Lachnospiraceae* and *Ruminococcaceae*, while reducing potentially harmful bacteria *e.g. Klebsiella*. These microbial shifts were accompanied by SCFAs production and the enrichment of metabolic pathways related to bile acids and lipids. This indicates that PZMP3 not only supports saccharolytic activity but also may influence host-bile acid interactions, which are linked to lipid digestion-thereby contributing to the regulation of host metabolism. Compared with inulin, which primarily enhanced classical probiotics and carbohydrate metabolism, the metabolic pathways enriched upon PZMP3 fermentation suggest the potential for broad functional implications, suggesting a potential route for immune modulation *via* microbial metabolites and this requires validation in future *in vivo* studies. The dual effects on microbial community structure and metabolic capacity highlight the multifunctional potential of PZMP3 as a novel dietary ingredient. These findings could provide a mechanistic foundation for its application in functional food development and suggest promising opportunities for translating jujube polysaccharides into health-promoting products targeting gut microbiota and metabolic homeostasis.

## CRediT authorship contribution statement

**Xiaoqiong Li:** Writing – original draft, Visualization, Formal analysis, Data curation, Conceptualization, Project management. **Ke Jiang:** Writing – original draft, Visualization, Formal analysis, Data curation, Conceptualization. **Huowang Zheng:** Investigation, Data curation. **Chenyu Zhao:** Validation, Formal analysis. **Jinjun Li:** Validation, Formal analysis. **Yuling Li:** Validation, Formal analysis. **Xiangyu Bian:** Validation, Formal analysis. **Jun Du:** Resources, Project management. **Liang Chen:** Resources, Project management. **Zhigang Zhu:** Resources, Project management. **Li Wang:** Resources, Project management. **Xiaolong Ji:** Writing – review & editing, Validation, Supervision, Resources. **Hongzhou Cui:** Funding acquisition, Writing – review & editing, Validation, Supervision, Resources.

## Ethics approval and consent to participate

All procedures received approval from the ethics committee of the pertinent departments at the Hangzhou Center for Disease Control and Prevention, and we have adhered to all applicable ethical guidelines.

## Funding

This work was carried out with the support of the National Natural Science Foundation of China (No. 32201969), “Pioneer” and eading Goose” R&D Program of Zhejiang (Grant No. 2025C02080), 10.13039/501100003819Natural Science Foundation of Hubei Province (2023AFB439), State Key Laboratory for Quality and Safety of Agroproducts (10417000025CE0632G), and Basic Research Program of Shanxi Province (Free Exploration Project No. 202303021221224).

## Declaration of competing interest

The authors declare that they have no known competing financial interests or personal relationships that could have appeared to influence the work reported in this paper.

## Data Availability

No data was used for the research described in the article.

## Appendix A Supplementary data

Table S1 Changes in total and reducing sugar content of PZMP3 after digestion and fermentation for 24 h. Supplementary material 1

Table S2 Results of differential metabolite screening in PZMP3 during fermentation. Supplementary material 2
