# Supplementary material for: In vitro digestion and fermentation of jujube-derived polysaccharide PZMP3 and its modulatory effect on gut microbiota and metabolic pathways
Source: Food Chem X. 2025 Nov 29;33:103316. doi: 10.1016/j.fochx.2025.103316 (PMC12764440; doi:10.1016/j.fochx.2025.103316)
Supplement: Table S1 Changes in total and reducing sugar content of PZMP3 after digestion and fermentation for 24 h. [file mmc1.docx]

Table S1 Changes in total and reducing sugar content of PZMP3 after digestion and fermentation for 24 h.

|  | total sugar (mg/mL) | reducing sugar (mg/mL) |
| --- | --- | --- |
| gastroenteric digestion | 0.220±0.007^b^ | 1.306±0.020^b^ |
| fermentation for 24 h | 0.006±0.001^a^ | 0.863±0.008^a^ |

Different lowercase letters in the same column indicate a different significance between treatments (*P* <0.05).
