# Supplementary material for: In vitro digestion and fermentation of jujube-derived polysaccharide PZMP3 and its modulatory effect on gut microbiota and metabolic pathways
Source: Food Chem X. 2025 Nov 29;33:103316. doi: 10.1016/j.fochx.2025.103316 (PMC12764440; doi:10.1016/j.fochx.2025.103316)
Supplement: Table S2 Results of differential metabolite screening in PZMP3 during fermentation. [file mmc2.docx]

Table S2 Results of differential metabolite screening in PZMP3

| Ion mode | Name | Formula | log^2^FC(P/B) | *P*-value | VIP |
| --- | --- | --- | --- | --- | --- |
| Negative | Ursodeoxycholic acid | C_24_H_40_O_4_ | 3.16 | 1.16E-04 | 30.06 |
| Negative | Cholesterol 3-sulfate | C_27_H_46_O_4_S | -3.68 | 3.54E-02 | 27.66 |
| Negative | Cholic acid | C_24_H_40_O_5_ | 6.62 | 6.34E-09 | 23.16 |
| Negative | Taurocholic acid | C_26_H_45_NO_7_S | 8.19 | 1.35E-05 | 15.96 |
| Negative | Stercobilinogen | C_33_H_48_N_4_O_6_ | -4.80 | 2.86E-08 | 13.41 |
| Negative | 12,13-DiHOME | C_18_H_34_O_4_ | 5.97 | 1.67E-06 | 5.28 |
| Negative | 5beta-Cyprinolsulfate | C_27_H_48_O_8_S | 7.71 | 5.30E-06 | 5.12 |
| Negative | Cucurbitacin B | C_32_H_46_O_8_ | 6.71 | 2.15E-06 | 3.58 |
| Negative | Zidovudine | C_10_H_13_N_5_O_4_ | -1.91 | 2.11E-03 | 2.99 |
| Negative | Cholestane-3,7,12,25-tetrol-3-glucuronide | C_33_H_56_O_10_ | -2.67 | 2.59E-04 | 2.04 |
| Negative | Deacetylcucurbitacin C | C_30_H_46_O_7_ | 7.16 | 6.56E-05 | 1.63 |
| Negative | Mesobilirubinogen | C_33_H_44_N_4_O_6_ | -4.13 | 4.16E-04 | 1.45 |
| Negative | 20-hydroxyecdysone | C_27_H_44_O_7_ | 4.26 | 1.01E-05 | 1.42 |
| Negative | L-Proline | C_5_H_9_NO_2_ | 6.28 | 1.68E-06 | 1.37 |
| Negative | Uridine 5'-diphosphoglucuronic acid | C_15_H_22_N_2_O_18_P_2_ | -1.08 | 5.57E-04 | 1.33 |
| Negative | Coenzyme Q10 | C_59_H_90_O_4_ | 2.79 | 3.14E-03 | 1.24 |
| Negative | Mycolactone | C_44_H_70_O_9_ | 7.30 | 2.80E-04 | 1.20 |
| Negative | 20-hydroxyecdysone | C_27_H_44_O_7_ | 5.50 | 1.80E-06 | 1.15 |
| Negative | 1-Octadecanoyl-2-(9Z,12Z-octadecadienoyl)-sn-glycero-3-phosphate | C_39_H_73_O_8_P | 3.65 | 2.21E-05 | 1.14 |
| Negative | Panthenol | C_9_H_19_NO_4_ | 1.09 | 1.66E-02 | 3.88 |
| Positive | Stercobilinogen | C_33_H_48_N_4_O_6_ | -6.71 | 3.42E-07 | 14.75 |
| Positive | D-erythro-C18-Sphingosine | C_18_H_37_NO_2_ | 8.20 | 2.93E-05 | 7.72 |
| Positive | Cholic acid | C_24_H_40_O_5_ | 8.35 | 5.76E-07 | 5.25 |
| Positive | Styrene | C_8_H_8_ | -4.08 | 1.19E-05 | 3.80 |
| Positive | L-Proline | C_5_H_9_NO_2_ | 5.78 | 1.42E-07 | 3.75 |
| Positive | L-Histidinol | C_6_H_11_N_3_O | -1.65 | 1.57E-03 | 3.58 |
| Positive | Taurocholic acid | C_26_H_45_NO_7_S | 7.38 | 1.73E-08 | 3.56 |
| Positive | Tyramine | C_8_H_11_NO | -1.61 | 3.47E-02 | 3.46 |
| Positive | Fluorene | C_13_H_10_ | 6.74 | 5.72E-05 | 2.72 |
| Positive | alpha-Linolenic acid | C_18_H_30_O_2_ | 5.20 | 1.57E-06 | 2.66 |
| Positive | 1-Naphthylmethanol | C_11_H_10_O | 7.46 | 6.97E-06 | 2.33 |
| Positive | Phytosphingosine | C_18_H_39_NO_3_ | -1.07 | 3.50E-03 | 2.27 |
| Positive | Indole | C_8_H_7_N | 1.89 | 3.13E-03 | 2.23 |
| Positive | D-erythro-C18-Sphingosine | C_18_H_37_NO_2_ | 4.12 | 1.40E-04 | 2.12 |
| Positive | Zymosterol | C_27_H_44_O | 8.10 | 3.46E-05 | 2.03 |
| Positive | 2-Polyprenylphenol | C_16_H_22_O | 10.75 | 1.48E-08 | 1.96 |
